# Serum iron levels as a new biomarker in chemotherapy with leucovorin and fluorouracil plus oxaliplatin or leucovorin and fluorouracil plus irinotecan, with or without molecularly-targeted drugs

**DOI:** 10.3892/mco.2013.136

**Published:** 2013-05-30

**Authors:** TAKUMI OCHIAI, KAZUHIKO NISHIMURA, TOMOO WATANABE, MASAYUKI KITAJIMA, AKINORI NAKATANI, TAKASHI INOU, HIDEKI SHIBATA, TSUYOSHI SATO, KENJI KISHINE, SHOUGO SEO, SATOSHI OKUBO, SHUNJI FUTAGAWA, SATOMI MASHIKO, ISAO NAGAOKA

**Affiliations:** 1 Department of Surgery, Tobu Chiiki Hospital, Tokyo Metropolitan Health and Medical Treatment Corporation, Tokyo 125-8512, Japan; 2 Department of Pharmacy, Tobu Chiiki Hospital, Tokyo Metropolitan Health and Medical Treatment Corporation, Tokyo 125-8512, Japan; 3 Department of Host Defense and Biochemical Research, Juntendo University School of Medicine, Tokyo 113-8421, Japan

**Keywords:** serum iron levels, biomarker, chemotherapy, leucovorin and fluorouracil plus oxaliplatin, leucovorin and fluorouracil plus irinotecan

## Abstract

Serum iron levels have been reported to increase following the administration of various anticancer drugs. An increase in serum iron levels during therapy with leucovorin and fluorouracil plus oxaliplatin (FOLFOX) or leucovorin and fluorouracil plus irinotecan (FOLFIRI) was also observed. The aim of this study was to investigate the correlation between serum iron levels and prognosis in advanced colorectal cancer (CRC) patients treated with FOLFOX/FOLFIRI ± molecularly-targeted drugs. Serum iron levels were measured prior to and at 48 h after treatment with FOLFOX/FOLFIRI ± molecularly-targeted drugs in 72 advanced CRC patients, all of whom succumbed to the disease between December, 2005 and February, 2012. No patients received radiotherapy. Taking the median rate of increase in serum iron levels as the cut-off value in each therapy, the patients were divided into cohort I (increase rate greater than the cut-off value in at least one therapy) or cohort II (increase rate less than the cut-off value in all therapies). The χ^2^ test and the t-test were used to compare patient characteristics between the two cohorts. Prognosis was evaluated between the two cohorts using the Kaplan-Meier method, the log-rank test and the Cox proportional hazards regression analysis. No significant bias in patient characteristics (including the frequency of chemotherapy or number of patients treated with molecularly-targeted drugs) was observed between the two cohorts. Serum iron levels were transiently elevated following treatment (P<0.001), returning to baseline within 2 weeks. Median survival time (MST) in cohort I (n=44) and cohort II (n=28) was 430 and 377 days, respectively. The MST was significantly higher in cohort I (P=0.0382). The multivariate analysis identified a small increase in serum iron levels as an independent risk factor for overall survival (OS). These results suggest that serum iron levels may be used as a new predictive factor in FOLFOX/FOLFIRI ± molecularly-targeted drug therapy. Serum iron levels may therefore prove to be a useful and convenient biomarker for OS in CRC patients.

## Introduction

The prediction of the host response to an administered therapy by means of a serum biomarker may offer a useful and convenient prognostic or predictive factor in the planning of cancer treatment. Follézou and Bizon (1) reported an increase in serum iron levels following administration of various anticancer drugs, including 5-FU, actinomycin D, adriamycin and cyclophosphamide. Recently, we also reported a significant increase in serum iron levels during therapy with leucovorin and fluorouracil plus oxaliplatin (FOLFOX) or leucovorin and fluorouracil plus irinotecan (FOLFIRI). Moreover, the levels of aspartate aminotransferase, alanine aminotransferase and hemoglobin were unaffected and the levels of transferrin and ferritin were only minimally altered during chemotherapy, while a molecularly-targeted drug exerted no effect on serum iron levels (2).

The aim of this study was to investigate the correlation between serum iron levels and prognosis in advanced colorectal cancer (CRC) patients treated with FOLFOX/FOLFIRI ± molecularly-targeted drugs, in order to establish their potential as a new biomarker.

## Patients and methods

### Patients

Seventy-two patients with unresectable advanced or metastatic CRC were enrolled in this study. Treatments based on the Japanese Society for Cancer of the Colon and Rectum guidelines were administered to all the patients at our institution (3). Patients were treated with FOLFOX or FOLFIRI therapy alone or in combination with molecularly-targeted drugs (bevacizumab/cetuximab/panitumumab). All patients succumbed to their disease between December, 2005 and February, 2012. No patients received radiotherapy. Informed consent for the measurement of serum iron levels was obtained from the patients. Approval for this study was obtained from the Tobu Chiiki Hospital Institutional Review Board (no. 12.09.10. no. 2).

### Serum iron levels

Serum iron levels were measured as part of routine blood analysis at our hospital laboratory prior to and 48 h after chemotherapy, to determine whether an adverse reaction had occurred. The normal range of serum iron levels was established as 60–210 μg/dl for men and 50–170 μg/dl for women. Changes in serum iron levels during chemotherapy were assessed. Taking the median rate of increase in serum iron levels as the cut-off value in each therapy, the patients were divided into two cohorts: cohort I (increase rate greater than the cut-off value in at least one therapy) or cohort II (increase rate less than the cut-off value in all therapies). Prognosis was prospectively evaluated and compared between the two cohorts.

### Statistical analysis

Patient characteristics were compared between the two cohorts using the χ^2^ test (gender, number of patients treated with molecularly-targeted drugs, Dukes’ stage, histological type, primary tumor site and recurrence type) and the t-test (age and frequency of chemotherapy). The median survival time (MST) by cut-off value of serum iron levels was calculated by the Kaplan-Meier method. The overall survival (OS) curves of the two cohorts as determined by the cut-off value were compared by the log-rank test. The Cox proportional hazards regression analysis was used in the univariate and multivariate analyses of prognostic factors for OS. P<0.05 was considered to indicate a statistically significant difference. Data were expressed as the means ± SD and were analyzed using SPSS for Windows version 15 (SPSS Inc., Chicago, IL, USA).

## Results

### Patient characteristics and serum iron levels

**Figure 1:**
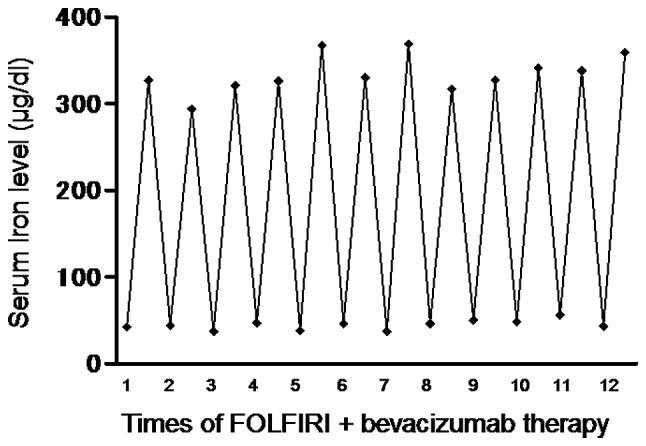
Typical pattern change of serum iron levels prior to and following therapy with leucovorin and fluorouracil plus irinotecan (FOLFIRI) + bevacizumab.

**Figure 2:**
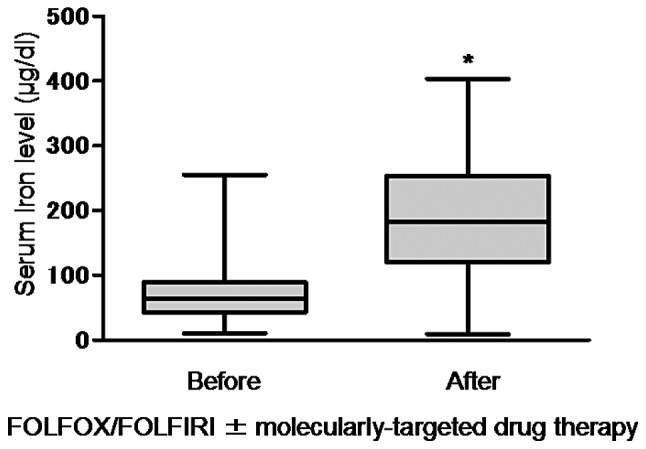
Change in serum iron levels prior to and following therapy with leucovorin and fluorouracil plus oxaliplatin (FOLFOX)/leucovorin and fluorouracil plus irinotecan (FOLFIRI) ± molecularly-targeted drug. Serum iron levels were measured in 1,454 blood samples. Data are presented as the means ± SD; values prior to and following chemotherapy were compared. ^*^P<0.001.

The patient characteristics are shown in Table I. The typical pattern of change in the serum iron levels prior to and following each chemotherapy regimen is shown in Fig. 1. The serum iron levels were transiently elevated following treatment, returning to baseline within 2 weeks. The serum iron level was 68.16±32.46 μg/dl prior to treatment, increasing significantly to 185.87±80.11 μg/dl following treatment (1,454 blood samples, P<0.001, Fig. 2). The median increase rate in the serum iron levels (cut-off value) is shown in Table II. No significant bias in patient characteristics was observed between cohorts I (n=44) and II (n=28) (Table III).

### Prognosis

**Figure 3:**
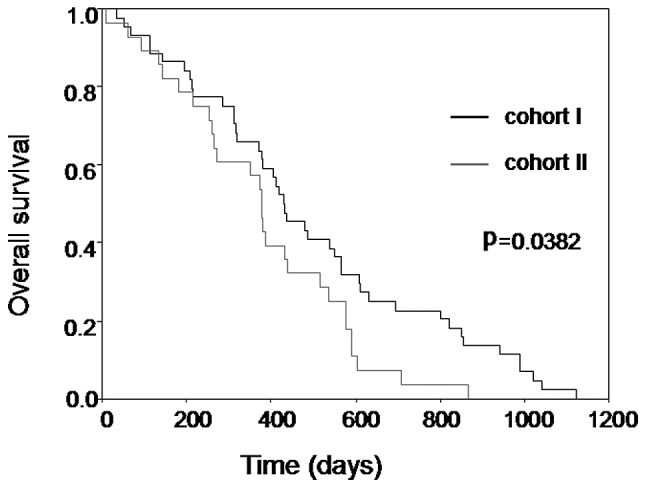
Overall survival rates in cohorts I (black line) and II (gray line).

The MST in cohorts I (n=44) and II (n=28) was 430 and 377 days, respectively. The MST was significantly higher in cohort I (P=0.0382) (Fig. 3). The results of univariate analysis are shown in Table IV. A significant difference was observed in the serum iron levels. Multivariate analysis identified a small increase in the serum iron levels as an independent risk factor for OS (Table V).

## Discussion

CRC is the third most common type of cancer worldwide and the fourth most common cause of cancer-related mortality (4). The OS rate in advanced CRC patients has increased over the past decade as a result of advances in chemotherapy. An increase in serum iron levels with the administration of various anticancer drugs was first reported several decades ago. We recently reported a significant increase in serum iron levels during FOLFOX or FOLFIRI therapy (2). In the present study, serum iron levels were investigated as a potential new biomarker of prognosis in chemotherapy.

Biomarkers play an important role in cancer diagnosis, prognosis, treatment and monitoring. Several biomarkers have been investigated with the development of new molecular biological techniques and advances in cancer biology. Preoperative increases in the serum levels of carcinoembryonic antigen, C-reactive protein (CRP), pro-inflammatory cytokine interleukin-6 (IL-6) and other markers have been reported to provide prognostic information (5–16). Prognostic factors for human CRC have been the focus of extensive investigation (17–19). Serum biomarkers have attracted attention as they offer a minimally invasive and convenient tool for determining prognosis. Serum iron levels are determined during the course of routine blood analysis. Therefore, they are a potential easy-to-use biomarker for chemotherapy in advanced CRC patients.

Iron is essential to all human cells, playing an important role in numerous biological processes, such as electron and oxygen transport and DNA synthesis (20,21). However, excess iron poses a threat to cells and tissues due to its ability to catalyze the generation of reactive radicals (22). Therefore, serum iron levels are strictly regulated in the human body (23), mainly by the peptide hepcidin, which is produced in the liver (24–26). Hepcidin is a key regulator of the metabolism of iron, controlling absorption and recycling (27,28). Hepcidin decreases intestinal iron absorption and increases its retention in reticuloendothelial cells (26). The target of hepcidin action is the iron exporter ferroportin, which is mainly present in the basolateral membranes of enterocytes and the cell membranes of macrophages and hepatocytes (29). Hepcidin is increased by iron loading and IL-6 and decreased by anemia or hypoxia (27,30–33). The majority of the iron required by the bone marrow for erythropoiesis is provided by recycling iron from senescent red blood cells via macrophages.

In this study, serum iron levels were transiently elevated following chemotherapy, returning to baseline within 2 weeks. A number of factors may have contributed to this phenomenon. Erythropoiesis, which consumes the largest amount of iron in the body, exerting a profound effect on its distribution and metabolism, is suppressed by chemotherapy. The subsequent reduction in iron consumption for hemoglobin synthesis may have caused this transient increase in serum iron levels. Vokurka *et al*(34) observed an increase in the expression of hepcidin associated with the irradiation-induced suppression of erythropoiesis in mice. Continuous iron absorption in the gut and its release from macrophages is highly undesirable in situations where erythropoiesis is suppressed. Moreover, the increase in the expression of hepcidin was observed even in the presence of severe anemia due to inhibition of hematopoiesis by irradiation. Hemolysis and anemia decrease hepcidin expression only when erythropoiesis is functional. However, if erythropoiesis is arrested, even severe anemia does not lead to a decrease in hepcidin expression, which is significantly increased. Hepcidin expression during chemotherapy was not measured in the present study. However, if such an increase in the expression of hepcidin was triggered by chemotherapy, the underlying mechanism may be similar to that induced by irradiation.

There were several limitations to this study. First, the patient sample was limited. A larger patient sample may improve data quality. Second, although serum iron levels appear to be a biomarker for OS, the correlation between the increase in serum iron levels and prognosis has not been fully elucidated, nor has that between increases in CRP or IL-6 and prognosis. In addition to cancer cells, chemotherapy suppresses erythropoiesis. If an increase in serum iron levels is the result of suppression of erythropoiesis, this may also indicate suppression of cancer cell proliferation. Third, neither hepcidin as a key regulator of iron metabolism nor IL-6 as a main inducer of hepcidin expression were investigated in the present study. A study on a larger patient population is currently being planned to investigate the association of systemic iron metabolism with the clinical outcome of chemotherapy.

In conclusion, no significant difference was observed in the frequency of chemotherapy or the number of patients treated with molecularly-targeted drugs between the two cohorts. Cohort I exhibited a statistically significant improvement in prognosis. Furthermore, the multivariate analysis revealed that the change in serum iron levels was an independent predictive variable. These results suggest that serum iron levels may be a useful and convenient biomarker for OS in CRC patients.

## Figures and Tables

**Table I:** Patient characteristics.

Variables	Value
No. of patients	72
Age in years [mean, (range)]	70.1 (45–84)
Gender (male/female)	41/31
Histological type	
Adenocarcinoma	
Well-differentiated	8
Moderately-differentiated	50
Poorly-differentiated	4
Mucinous carcinoma	8
Unknown	2
Primary cancer site	
Colon/rectum	54/18
Dukes’ stage (A/B/C/D)	1/6/35/30
Recurrence type	
Lymph node	4
Liver	33
Local recurrence	3
Bone	2
Mediastinum	1
Lung	12
Unresectable	7
Peritoneum	9
Ovary	1
Molecularly-targeted drug (+/−)	29/43
Frequency of chemotherapy (range)	21.5 (1–73)

**Table II:** Median increase rate in serum iron levels (cut-off values).

Parameters	FOLFOX4 (n=96)	mFOLFOX6 (n=4)	FOLFIRI (n=69)
Alone	214.2% (n=65)	577.3% (n=2)	344.3% (n=41)
Molecularly-targeted drug	190.2% (n=31)	501.6% (n=2)	395.3% (n=28)

FOLFOX, leucovorin and fluorouracil plus oxaliplatin; FOLFOX4, day 1: oxaliplatin 85 mg/m^2^, L-leucovorin 100 mg/m^2^ (L-isomer form), fluorouracil bolus 400 mg/m^2^, fluorouracil infusion 600 mg/m^2^ for 22 h, day 2: L-leucovorin 100 mg/m^2^, fluorouracil bolus 400 mg/m^2^, fluorouracil infusion 600 mg/m^2^ for 22 h; mFOLFOX6, oxaliplatin 85 mg/m^2^, L-leucovorin 200 mg/m^2^, fluorouracil bolus 400 mg/m^2^, fluorouracil infusion 2,400 mg/m^2^ over 46 h; FOLFIRI, leucovorin and fluorouracil plus irinotecan; n, number of patients.

**Table III:** Patient characteristics.

Variables	Cohort I	Cohort II	P-value
No. of patients	44	28	
Age in years [mean, (range)]	71.8 (53–84)	67.5 (45–81)	0.062
Gender (male/female)	27/17	14/14	0.464
Histological type			0.181
Adenocarcinoma			
Well-differentiated	4	4	
Moderately-differentiated	30	20	
Poorly-differentiated	3	1	
Mucinous carcinoma	7	1	
Unknown	0	2	
Primary cancer site			
Colon/rectum	34/10	20/8	0.463
Dukes’ stage (A/B/C/D)	1/3/24/16	0/3/11/14	0.470
Recurrence type			0.096
Lymph node	3	1	
Liver	19	14	
Local recurrence	3	0	
Bone	0	2	
Mediastinum	0	1	
Lung	10	2	
Unresectable	2	5	
Peritoneum	6	3	
Ovary	1	0	
Molecularly-targeted drug (+/−)	21/23	8/20	0.141
Frequency of chemotherapy (range)	23.6 (1–73)	18.3 (1–41)	0.113

**Table IV:** Univariate analysis of overall survival.

Variables	No. of patients	Hazard ratio	95% CI	P-value
Age (years)		0.964	0.574–1.620	0.891
<75	51			
75≤	21			
Gender		0.622	0.378–1.022	0.061
Male	42			
Female	30			
Histological type		0.657	0.364–1.188	0.164
Differentiated	58			
Undifferentiated/unknown	14			
Primary site		0.720	0.417–1.242	0.238
Colon	54			
Rectum	18			
Dukes’ stage		1.236	0.764–1.999	0.388
A/B/C	42			
D	30			
Recurrence type		1.767	0.894–3.494	0.102
Local recurrence/Unresectable	10			
Metastasis	62			
Serum iron level		1.686	1.023–2.779	0.040
Cohort I	44			
Cohort II	28			

CI, confidence interval.

**Table V:** Multivariate analysis of overall survival.

Variables	No. of patients	Hazard ratio	95% CI	P-value
Age (years)		1.216	0.664–2.229	0.526
<75	51			
75≤	21			
Gender		0.561	0.297–1.061	0.075
Male	42			
Female	30			
Histological type		0.589	0.285–1.219	0.154
Differentiated	58			
Undifferentiated/unknown	14			
Primary site		0.731	0.389–1.375	0.331
Colon	54			
Rectum	18			
Dukes’ stage		1.333	0.789–2.250	0.283
A/B/C	42			
D	30			
Recurrence type		2.096	0.960–4.575	0.063
Local recurrence/unresectable	10			
Metastasis	62			
Serum iron level		1.961	1.143–3.365	0.015
Cohort I	44			
Cohort II	28			

CI, confidence interval.
